# Enantioselective and Synergistic Herbicidal Activities of Common Amino Acids Against *Amaranthus tricolor* and *Echinochloa crus-galli*

**DOI:** 10.3390/molecules26072071

**Published:** 2021-04-04

**Authors:** Nawasit Chotsaeng, Chamroon Laosinwattana, Patchanee Charoenying

**Affiliations:** 1 Department of Chemistry, School of Science, King Mongkut’s Institute of Technology Ladkrabang, Bangkok 10520, Thailand; patchanee.ch@kmitl.ac.th; 2 Integrated Applied Chemistry Research Unit, School of Science, King Mongkut’s Institute of Technology Ladkrabang, Bangkok 10520, Thailand; 3 Department of Plant Production Technology, School of Agricultural Technology, King Mongkut’s Institute of Technology Ladkrabang, Bangkok 10520, Thailand; chamroon.la@kmitl.ac.th

**Keywords:** herbicidal, inhibitory, synergistic, enantioselective, amino acids, d-isomer, racemic mixture, racemate, l-isomer, Chinese amaranth, barnyard grass

## Abstract

Amino acids have a wide range of biological activities, which usually rely on the stereoisomer presented. In this study, glycine and 21 common α-amino acids were investigated for their herbicidal property against Chinese amaranth (*Amaranthus tricolor* L.) and barnyard grass (*Echinochloa crus-galli* (L.) Beauv.). Both d- and l-isomers, as well as a racemic mixture, were tested and found that most compounds barely inhibited germination but moderately suppressed seedling growth. Various ratios of d:l-mixture were studied and synergy between enantiomers was found. For Chinese amaranth, the most toxic d:l-mixtures were at 3:7 (for glutamine), 8:2 (for methionine), and 5:5 (for tryptophan). For barnyard grass, *rac*-glutamine was more toxic than the pure forms; however, d-tryptophan exhibited greater activity than racemate and l-isomer, indicating the sign of enantioselective toxicity. The mode of action was unclear, but d-tryptophan caused bleaching of leaves, indicating pigment synthesis of the grass was inhibited. The results highlighted the enantioselective and synergistic toxicity of some amino acids, which relied upon plant species, chemical structures, and concentrations. Overall, our finding clarifies the effect of stereoisomers, and provides a chemical clue of amino acid herbicides, which may be useful in the development of herbicides from natural substances.

## 1. Introduction

It is well-known that to produce enough agricultural crops to feed the growing world population, farmers must rely on synthetic chemicals to eliminate pests and weeds. However, the use of these chemicals is like a double-edged sword, which has both advantages and disadvantages [1,2]. On the one hand, these herbicides allow us to achieve high crop yields. On the other hand, these chemicals, when left behind in the environment, are usually toxic to plants, animals, or even humans. Therefore, recently, agricultural scientists are looking for a new approach for weed control [3,4,5]. Many chemists believe that alternative herbicides should be natural products [6,7]. This concept is widely accepted because of the long-standing belief that natural substances are relatively safer than synthetic compounds. Also, they are likely to degrade quickly and not accumulate in the environment.

Since it has been discovered that some plants are harmful to other plants by releasing natural substances, called “allelochemicals”, into the environment, many chemists have investigated the effects of these compounds on weeds, both in controlled laboratory and field conditions [8,9,10,11]. Particularly, those works did not focus only on pure forms, but also crude extracts, or even mixtures of crushed plant samples [12]. Recently, this research has received increased attention, due to the ongoing evolution of organic agriculture.

Amino acids are molecules in which the chemical structure consists of amino and carboxyl groups. Generally, in biochemistry [13], amino acids often refer to a specific group—the alpha (α-) amino acids, compounds that containing both functional groups on the same carbon atom. In nature, protein chains of all living species are constructed by joining various combinations of about 20 natural α-amino acids [14,15]. Therefore, those compounds are crucially important to living creatures. Apart from being used as a food supplement, natural amino acids are also used for other purposes. For example, several research groups have reported that common amino acids or related compounds could show anticancer [16,17] and antimicrobial [18,19,20] activities or even prevent some diseases [21,22,23]. In addition to those medicinal properties, natural amino acids also show herbicidal activities against some plants [24,25,26,27,28,29,30,31,32]. For example, a study on the herbicidal potential of 20 common amino acids on a dicotyledonous parasitic plant, *Orobanche minor*, revealed that among the tested substances lysine, methionine, and tryptophan strongly inhibited the early development of the plant [25]. Although this provided us with useful information about the role of amino acids as herbicides, it was still limited to racemic mixtures and some d- or l-isomers [29]. The weed-control ability of both d- and l- isomers, as well as racemic mixtures of common amino acids, has rarely been compared.

**Figure 1:**
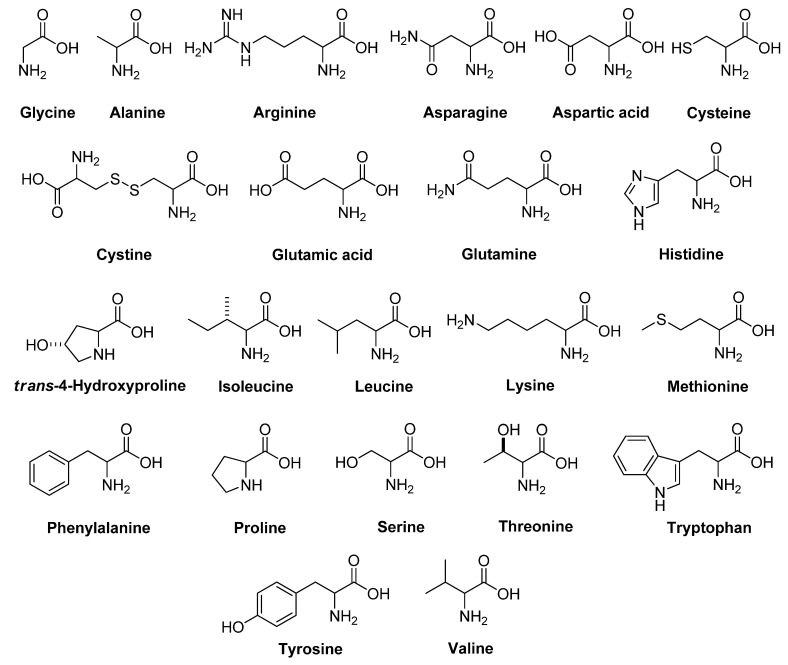
Common amino acids used in this study.

In general, a biological activity of active compounds relies on molecular conformations and absolute configurations [33]. Sometimes, both enantiomers of drug show similar pharmaceutical effects, but mostly the two isomers have different biological activities [34]. In this regard, the tissues of a living creature are responsible for the differences in biological effects. Normally, biological systems exist exclusively in one enantiomeric form, so that they will interact differently with the two enantiomers of active compounds [35,36]. Numerous herbicides have chiral structures, and the two enantiomers have different actions against target weeds [37,38,39,40,41]. Therefore, we studied the different herbicidal effects of isomers of common amino acids (Figure 1), together with their racemic mixtures, on representative monocot and dicot plants. We aimed to clarify types of active compounds and their configurations, which could be used in the development of natural herbicides for organic agriculture.

## 2. Results

### 2.1. Herbicidal Activity of Glycine and 21 Common Amino Acids on Chinese Amaranth

**Figure 2:**
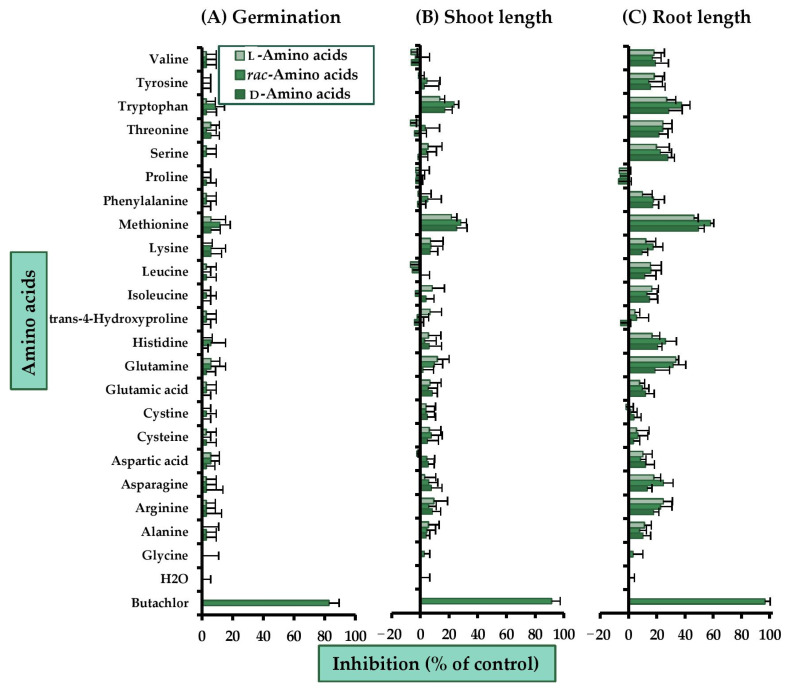
Inhibitory effects of amino acids at 2 mM on (**A**) seed germination, (**B**) shoot, and (**C**) root growth of Chinese amaranth. Butachlor and distilled water were used as positive and negative controls. Horizontal bars represent the standard error of an average of four replicates.

Glycine, together with another 21 common amino acids at concentration of 2 mM [29], were evaluated for herbicidal activity against a representative dicot plant, Chinese amaranth (*Amaranthus tricolor* L.) (Figure 2). The amino acids were used as single enantiomers and as racemic mixtures. All tested amino acids showed weaker inhibitory activity than commercial butachlor. For seed germination, only methionine and tryptophan showed a very small inhibition, but other amino acids had no activity. Also, racemic mixtures were slightly more harmful than the pure forms. For shoot growth, again, methionine and tryptophan had the highest inhibitory activity, and the racemic mixtures were still more effective than the d- and l-isomers. *rac*-Methionine inhibited shoot growth by ~28% and *rac*-tryptophan by ~24%. Most compounds inhibited root growth, and the activity level depended on absolute configurations and compound types. For some amino acids—i.e., aspartic acid, glutamic acid, serine, and valine—the d-isomers were a little more active than the racemic mixtures and l-forms. However, for most compounds—including asparagine, histidine, lysine, methionine, phenylalanine, and tryptophan—the mixtures had stronger inhibitory effects than the pure forms. For other amino acids—e.g., arginine, glutamine, isoleucine, threonine, tyrosine, and valine—the l-isomer was a little more active. Among 22 tested compounds, Chinese amaranth root elongation was suppressed by l-glutamine (34%), *rac*-methionine (58%), and *rac*-tryptophan (35%).

### 2.2. Herbicidal Activity of Glycine and 21 Common Amino Acids on Barnyard Grass

**Figure 3:**
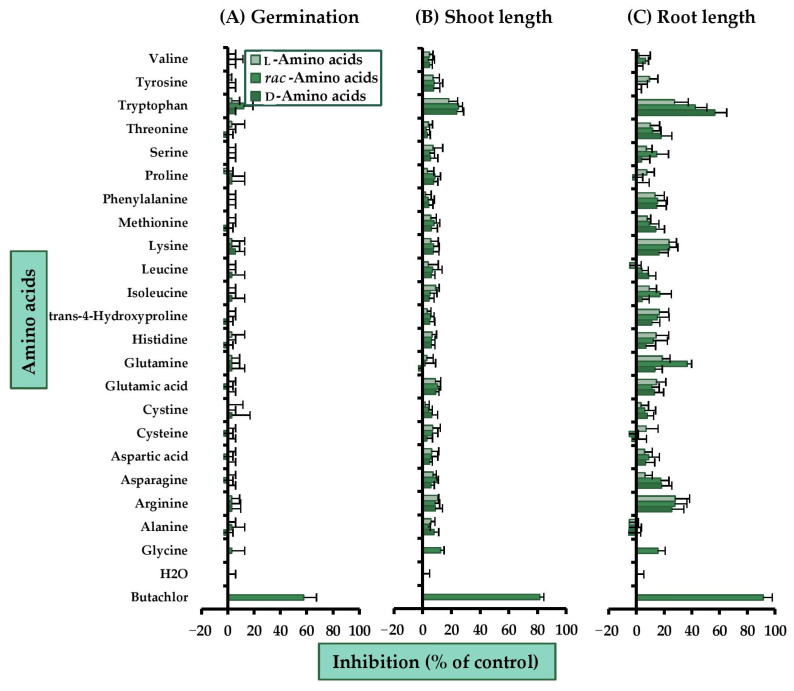
Inhibitory effects of amino acids at 2 mM on (**A**) seed germination, (**B**) shoot, and (**C**) root growth of barnyard grass. Butachlor and distilled water were used as positive and negative controls. Horizontal bars represent the standard error of an average of four replicates.

For a monocot species, barnyard grass (*Echinochloa crus-galli* (L.) Beauv.) (Figure 3), all tested amino acids had very low or no inhibitory activity compared to the commercial butachlor. Among these compounds, only *rac*-tryptophan was toxic to plant germination, and it very slightly inhibited seed germination of the plant (~12%). For shoot growth, again, nearly all tested substances had no significant effect on shoot elongation, except tryptophan, which moderately inhibited shoot length. At 2 mM, the tryptophan racemic mixture suppressed shoot extension by 25%. For root growth, it was clear that most amino acids had low to moderate herbicidal activities against root elongation, and sometimes the mixtures and individual isomers showed different levels of activity. Tryptophan was again the most active root inhibitor, with root elongation suppressed by 57% by the d-isomer, 43% by the racemic mixture, and 28% by the l-isomer. This indicated that, the d-form was more toxic than the others, and the effect of tryptophan was enantioselective. Apart from tryptophan, the basic amino acids, arginine, histidine, and lysine, also showed some harmful effects, especially the l-form and the mixture, which inhibited root length by 14–28%. Glutamine was the second most active compound against root growth. Its d-isomer, racemic mixture, and l-isomer restrained root growth by 13%, 37%, and 19% respectively. This was a sign of synergism among the enantiomers, where the mixture was the most active form.

### 2.3. Synergistic Effects of d- and l-Amino Acids on Tested Plants

**Figure 4:**
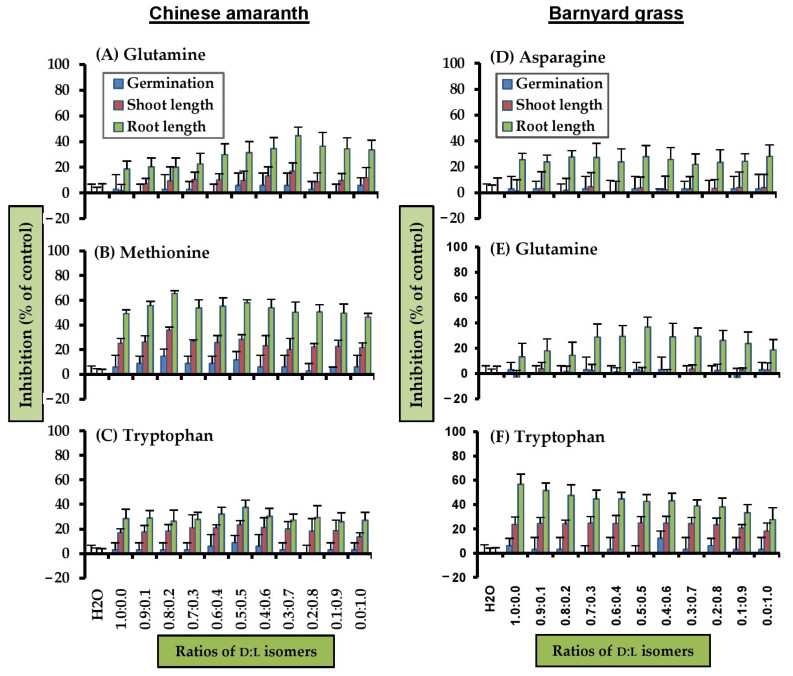
Inhibitory effects of mixtures of d- and l- amino acids at 2 mM on seed germination, shoot and root growth of Chinese amaranth and barnyard grass. Distilled water was the control. Vertical bars represent the standard error of an average of four replicates.

These results suggested that types of amino acids and isomers affected toxicity. To clarify this hypothesis, d-isomer, l-isomer, and different d:l mixtures of the most active substances, were tested against plant growth (Figure 4). Glutamine, methionine, and tryptophan were chosen for Chinese amaranth, and arginine, glutamine, and tryptophan were evaluated for barnyard grass.

#### 2.3.1. Chinese Amaranth

For Chinese amaranth, it was clear that all mixtures of d- and l-glutamine had no harmful activity on seed germination (Figure 4A). However, some mixtures were able to inhibit shoot length, where the l-isomer was more effective than the d-isomer, and 3:7 d:l-mixture had the highest inhibitory effect on shoot length (17%). Likewise, all mixtures highly inhibited root growth, with, in order, d-, l-, and 3:7 d:l ratio, being the most effective (45% inhibition).

Methionine (Figure 4B), all ratios, little inhibited seed germination of Chinese amaranth, with only the 8:2 ratio slightly inhibiting germination (~15%). However, for shoot growth, every mixture inhibited the shoot growth by more than 20%. The 8:2 d:l-mixture was still the best ratio, suppressing shoot growth by 36%. For all ratios, root growth was inhibited similarly, with 8:2 ratio suppressing root elongation by 65%. Thus, the 8:2 d:l ratio led to the greatest synergy.

For tryptophan (Figure 4C), clearly, all mixtures did not inhibit seed germination, but inhibited shoot growth by more than 17%. The racemic mixture was the most active, suppressing shoot growth by 24%. The harmful effect on root growth was similar that for shoot. All ratios suppressed root growth, but the racemic mixture was more effective, inhibiting root growth by 38%.

#### 2.3.2. Barnyard Grass

For barnyard grass, all mixtures of d- and l-asparagine were unable to inhibit seed germination (Figure 4D). However, all ratios clearly suppressed root growth at similar rate (22–28%). Although glutamine (Figure 4E) did not significantly inhibit seed germination and shoot growth, but it clearly suppressed root growth. Moreover, the l- was slightly more toxic than the d-isomer. All mixtures between d- and l-isomers showed synergy. Among those, the racemic mixture was more effective, inhibiting root growth by 37%.

Insignificant detrimental effect of tryptophan on seed germination was found (Figure 4F). However, for seedling growth, it slightly inhibited shoot length. The inhibitory effect on shoots was quite similar in almost all d- and l-mixtures, 21–25% inhibition, with the pure L-form exhibited weakest activity (18%). For root growth, the d-isomer suppressed root elongation the most (57%). The inhibition decreased when the ratio of the d-isomer decreased. Also, pure l-isomer was least active and reduced root growth by only 28%. Thus, d-tryptophan was more harmful than the mixture and l-tryptophan.

### 2.4. Dose Responses of Active Amino Acids on Chinese Amaranth

**Figure 5:**
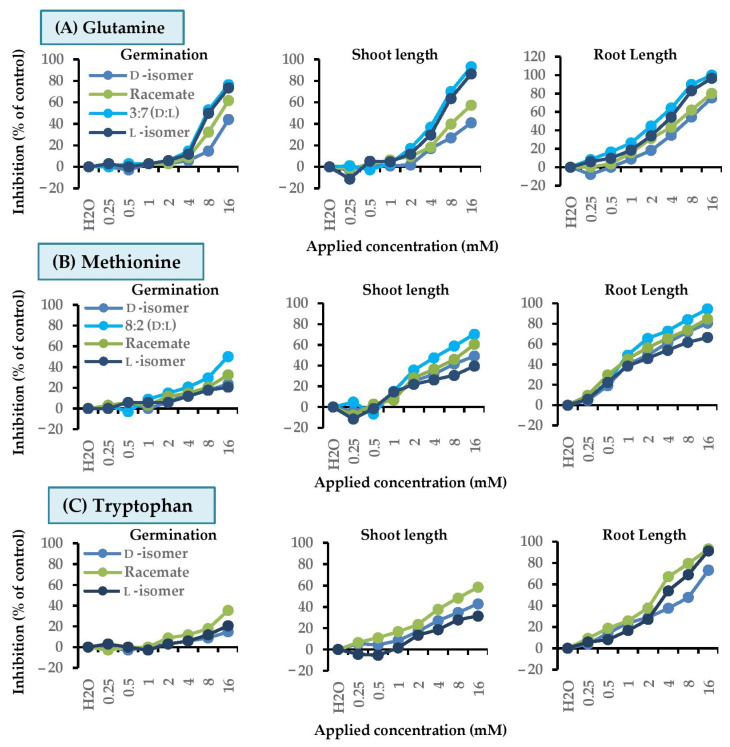
Inhibitory effects of d-isomer, l-isomer and d:l-mixtures of (**A**) glutamine, (**B**) methionine, and (**C**) tryptophan at 0.25–16 mM on seed germination, shoot and root growth of Chinese amaranth. Distilled water was used as a reference.

**Figure 6:**
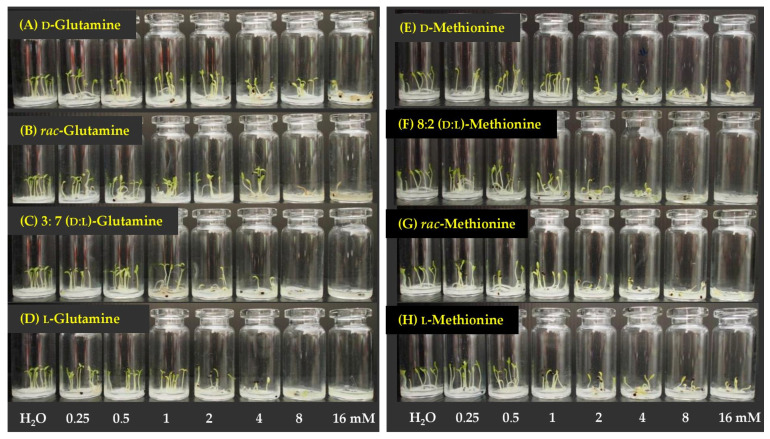
Inhibitory effects of d-isomer, l-isomer, and d:l-mixtures of glutamine (**A**–**D**) and methionine (**E**–**H**) at 0.25–16 mM on seed germination, shoot, and root growth of Chinese amaranth. Distilled water was used as a reference.

At 2 mM, we showed that various mixtures had different herbicidal activity. So here we measured the effect of concentrations on activity, ranging from 0.25 to 16 mM, with distilled water as a reference. For Chinese amaranth, three amino acids—glutamine, methionine, and tryptophan—were chosen. The results are shown in Figure 5 and Figure 6.

Glutamine, at concentrations of 0.25–2 mM, did not inhibit seed germination (Figure 5A and Figure 6A–D, but at higher concentrations, it showed greater activity. At 4–16 mM, 3:7 d:l-mixture and l-form were most effective, and suppressed plant germination by 15–77%. However, d-glutamine was least active, while racemate moderately affected plant germination. For shoot and root growth, 3:7 d:l-mixture and l- were still more toxic than the racemate and d-form, especially at high concentrations. At 16 mM, the mixture suppressed shoot elongation up to 93%, and completely inhibited root growth. These results clarified a little synergy between d- and l-glutamine against plant growth.

Regarding methionine, clearly, d:l-mixtures were more harmful than the pure forms Figure 5B and Figure 6E–H, especially the 8:2 d:l-mixture, which highly reduced germination (50%), shoot (70%), and root (95%) growth of the plant. However, the d- and l-isomers averagely suppressed Chinese amaranth growth. For tryptophan (Figure 5C), a racemic mixture significantly inhibited seed germination and seedling growth of the plant, and the effect was greater than that of pure isomers, particularly for shoot growth. These results indicated a synergistic toxicity between pure enantiomers of the amino acids.

Dose response results revealed that glutamine, methionine, and tryptophan, affected roots more than shoots, and the least impact was found on plant germination. In addition, the inhibition was concentration-dependent, where the weed control ability increased with the increase in concentration of the substances.

### 2.5. Dose Responses of Active Amino Acids on Barnyard Grass

**Figure 7:**
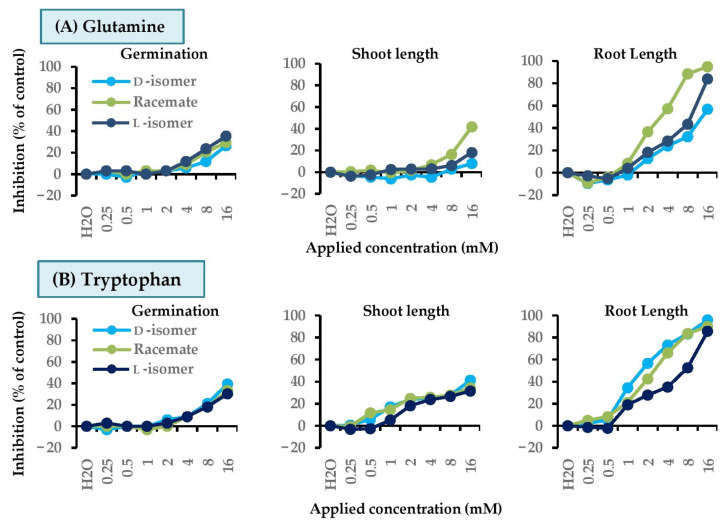
Inhibitory effects of mixtures of d-isomer, l-isomer and racemate of (**A**) glutamine and (**B**) tryptophan at 0.25–16 mM on seed germination, shoot, and root growth of barnyard grass. Distilled water was used as a reference.

**Figure 8:**
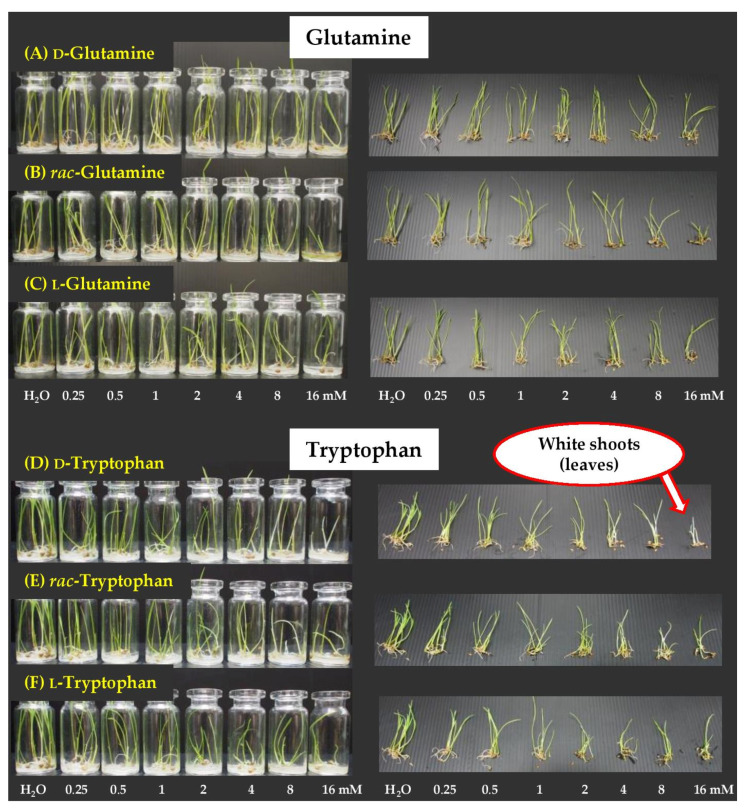
Inhibitory effects of mixtures of d-isomer, l-isomer, and racemate of glutamine (**A**–**C**) and tryptophan (**D**–**F**) at 0.25–16 mM on seed germination, shoot, and root growth of barnyard grass. Distilled water was used as a reference.

For barnyard grass (Figure 7 and Figure 8), we chose glutamine and tryptophan for further study, but not arginine, because glutamine showed a sign of synergistic effect towards root growth. Also, d-tryptophan was clearly more effective than the racemate and l-form, but all mixtures of arginine provided similar level of activity.

Both d- and l-, as well as *rac*-glutamine, had relatively similar activity against seed germination (Figure 7A and Figure 8A–C). However, for shoot growth, *rac*-glutamine was much more toxic than the pure forms, especially at high concentrations. At 16 mM, *rac*-glutamine suppressed shoot elongation by 42%, but d- and l-isomers exhibited weaker activity (~8% and 18%). Similarly, roots of the grass were more affected by *rac*-glutamine than by the pure enantiomers. At 4, 8, and 16 mM, this mixture suppressed root growth by 57%, 89%, and 95%. The results indicated synergistic behavior between d-glutamine and L-glutamine.

Herbicidal effect of tryptophan was showed in Figure 7B and Figure 8D–F. Racemic mixture and pure isomers exhibited similar activity against seed germination. However, for shoot growth, at low concentrations, the d- and racemate had a little stronger effect than l-form. In terms of root growth, L-isomer was less effective than other forms, while d- was most toxic. At 4, 8, and 16 mM, d-tryptophan suppressed root elongation by 66%, 84%, and 90%. The level of root inhibition of *rac*-tryptophan was in between the d- and l-form, but, at 4 and 8 mM, its effect was closer to that of d-isomer than l-isomer.

The harmful effect of tryptophan was determined not only by percent inhibition of seed germination and growth, but also by physical characteristics of the plant. It was revealed that D-tryptophan caused the color of shoots (or leaves) noticeably faded or bleached, especially at high concentrations (Figure 8D). *rac*-Tryptophan, at high concentrations, also showed this symptom (Figure 8E), but l-tryptophan did not decolor of shoots. This indicated that the d-form was more toxic to the plant than the l-, especially for the pigmentation in plants.

From dose–response curves, both glutamine and tryptophan more affected to roots than shoots and germination. Again, the herbicidal effect of these amino acids was dose-dependent, where the inhibition increased as the concentration of the compounds increased.

## 3. Discussion

Our preliminary screening revealed that some of the 22 common amino acids moderately inhibited shoot growth and highly suppressed root growth of both Chinese amaranth and barnyard grass. However, most of them weakly or not at all inhibited the germination. Similarly, Fernández-Aparicio et al. [42] investigated the inhibitory effects of allelochemicals identified in cereals (benzoxazolinones, hydroxycinnamic acids, l-tryptophan, and other compounds) on a parasitic plant, broomrapes (*Orobanche crenata*), and found that l-tryptophan strongly inhibited radicle growth, but it had no significant effect on germination. This is consistent with our results, indicating that common amino acids would potentially be active as a post-emergent rather than a pre-emergent herbicide, for certain species.

We also observed differential responses for shoots and roots. This is consistent with our earlier reports [43,44,45], that some natural and synthetic substances exhibited more harmful effect on roots of Chinese amaranth and barnyard grass than shoots. Comparably, Thi et al. [46] reported that root growth of barnyard grass was more sensitive to *N*-*trans*-cinnamoyltyramine herbicide than shoot growth. This was attributed to different permeabilities of active chemicals into roots and shoots, different enzyme profiles, plant organs, and growth stages.

Moreover, for some amino acids, the type of enantiomer clearly affected the level of inhibition. For example, d-tryptophan was more toxic to barnyard grass than the l-isomer or the mixture. The reason was unclear, in general, herbicidal activity of chiral compounds depends very much on their configuration [31,34,37]. Sometimes, only R-isomers are active or more active and sometimes only S-isomers are active. For instance, Zhang et al. [47] investigated the enantioselective damage of dicloflop on *Arabidopsis thaliana*, and found that R-isomer could show a greater toxicity than the racemate and S-isomer. Enantioselective activity of imazapyr [48] was reported against *A. thaliana* growth: (+)-imazapyr had a stronger herbicidal effect than *rac*-imazapyr and (−)-imazapyr. A freshwater algae, *Microcytis aeruginosa* [49], was also showed that (S)-metolachlor was significantly more harmful to algal growth, chlorophyll a content, and cell integrity of the algae, than any other isomers.

Previously, many reports [50] focused on plant responses to amino acids. For example, the effects of amino acids on barley (*Hordeum vulgare*) and peas (*Pisum sativum*) [51], duckweed (*Spirodela oligorrhiza*) [52], rice (*Oryza sativa*), and tobacco (*Nicotiana tabacum*) [53], showed that several d- isomers (e.g., alanine, histidine, and methionine) and some l-amino acids (e.g., histidine and methionine), reduced plant growth. Also, none of d-amino acids promoted duckweed growth, with d-Serine and d-alanine were most toxic among those tested D-isomers. Similarly, the ability of model plants (*Arabidopsis*) to use 15 amino acids (glycine, l-glutamine, l-asparagine, l-glutamic acid, l-aspartic acid, l-alanine, l-serine, l-arginine, l-valine, l-isoleucine, d-alanine, d-serine, d-arginine, d-valine, and d-isoleucine) as nitrogen sources was investigated [54]. Obviously, when applied as the sole nitrogen source, many l-amino acids could support plant growth, but nearly all d-forms inhibited plant development. Among those, d-isomers, d-alanine, and d-serine were absorbed by plant at the highest rates, and they were also the most effective growth inhibitors. This result supported their hypothesis that d-amino acids cannot be used for plant growth.

As documented above, usually, d-amino acids were more toxic to plants than the l-isomers, but Bertin et al. [24] found an opposite result. By the filter-paper bioassay, both d- and l-*m*-tyrosine showed equal toxicity against lettuce (*Lactuca sativa*) growth, but in an agar-based *Arabidopsis* bioassay, l-*m*-tyrosine was ten times stronger as an inhibitor than the D-form. This was believed to be the difference in uptake profiles and biochemical mechanisms of both enantiomers in the tested plants, which would be very complex. Similarly, Fernández-Aparicio et al. [25] suggested species, plant development stages, and experimental conditions, affected the herbicidal activity of amino acids. In in vitro experiments, lysine was the most toxic to the parasitic plant, *Orobanche minor*, followed by methionine and tryptophan; however, in field studies, methionine was most effective against plant emergence.

In our work, both isomers of some active amino acids showed a synergy as a herbicide. For instance, the effect on Chinese amaranth, 3:7 d:l mixture of glutamine, 8:2 d:l mixture of methionine, and *rac*-tryptophan, were more toxic than pure isomers. Similarly, for the monocot grass, *rac*-glutamine had greater activity than both pure forms. These results indicated that synergy was crucial for growth inhibition. Similarly, Ye et al. [41] investigated the enantioselective physiological effects of diclofop on cyanobacterium, *Microcystis aeruginosa*. They found that *rac*-dicoflop inhibited protein production and biomass growth, while both R- and S-isomers stimulated. It was believed that the synergy resulted from the in vivo enantiomerization in organisms, or the differences in toxicity mechanisms, where one isomer facilitated or hindered the other from binding to the active sites.

Apart from inhibiting plant growth, d-tryptophan also caused bleaching in plant leaves, especially at high concentrations. Although l-isomer did not show this symptom, the racemic mixture had a similar sign of injury. The reason was unclear, but we believed that the d-form induced leaf bleaching by inhibiting pigment synthesis. There are many modes of action [36,55,56] of herbicides, which often show different symptoms. Pigment inhibitors disrupt biosynthetic pathways, leading to leaf bleaching, and eventually plant death [36,55].

Our current study revealed that certain compounds (e.g., glutamine, methionine, tryptophan, arginine, histidine and lysine) inhibited plant growth. These results were in agreement with the previous reports [25,29], showing that arginine, lysine, methionine and tryptophan affected broomrape growth. Also, we found that substituents had a significant impact on herbicidal property. All basic amino acids, namely arginine, lysine and histidine, moderately inhibited root elongation. A trend of activity relied on the basicity, which the most basic arginine highly inhibited plant root. Besides, methionine, a thioether-substituted compound, was more toxic than thiol (cysteine) or disulfide (cystine). These results together with our previous work [45] highlighted the significant effect of sulfur-containing compounds on herbicidal property. For amidic amino acids, the longer-chain glutamine affected plant growth more than the shorter-chain asparagine. It was not fully understood, but the size of hydrocarbon chain was crucial for activity. For amino acids with aromatic rings, tryptophan, an indole containing compound, affected plant growth the most. Previously [57], some indole amides strongly inhibited radicle and shoot growths of lettuce (*Lactuca sativa*) and onion (*Allium cepa*). The effect was structure and concentration dependent, where at low concentrations, some derivatives stimulated plant growth. A series of indoles were also evaluated as plant growth inhibitors [58], some derivatives averagely suppressed germination and growth of *Ipomoea grandifolia*. Moreover, a research on *Piriformospora indica*-barley symbiosis revealed that indoles, elicited by root endophyte, *P. indica*, are not required for growth promotion but for colonization of barley root [59]. Apart from those mentioned groups, other substituents—i.e., alkyl, carboxyl, and hydroxyl—did not affect or slightly affected the tested species. Our results emphasized the importance of substituents on activity.

Another significant factor, affecting herbicide efficacy, was concentration. Herbicides are usually more effective at high concentrations, but at low concentrations they are less effective, instead, they become a growth-promoter [60,61]. Similar to our work, at high concentrations, amino acids inhibited plant growth, but at low concentrations, some of them slightly promoted plant development. In addition, we demonstrated that tested plants response differently to amino acids. Chinese amaranth was usually more susceptible than barnyard grass, in other words, the herbicides were more toxic to Chinese amaranth than the grass. It is common that the responses of plants to chemicals depend on physiological and biochemical properties of each species [62].

Factors that cause amino acids to show different inhibitory levels are not yet fully understood, but these herbicides may have different capabilities to bind to the molecular target sites of the plants. From a wider perspective, the amino acids would have different mechanisms within plant organisms, and these modes of action could be very complex and require further investigation.

## 4. Materials and Methods

### 4.1. Chemicals

Glycine and all 21 amino acids (both d- and l isomers) were purchased from Tokyo Chemical Industry (TCI, Tokyo, Japan) and Sigma-Aldrich (Singapore). *rac*-Amino acids and other ratios of d:l-mixture were prepared by mixing the solutions of d- and l-isomers in the required ratios. Butachlor (or *N*-(butoxymethyl)-2-chloro-*N*-(2,6- diethylphenyl)acetamide) was available from Sinon Corporation (Bangkok, Thailand).

### 4.2. Tested Plants

Similar to our previous report [44], Chinese amaranth (*Amaranthus tricolor* L.) and barnyard grass (*Echinochloa crus-galli* (L.) Beauv.) were used as representative dicotyledon and monocotyledon plants. Seeds of the former were purchased from Thai Seed & Agriculture Co. Ltd. (Bangkok, Thailand), and seeds of the latter were collected from rice field in the northern part of Thailand (Phitsanulok), in December 2019. Both species have a germination rate around 85–90%.

### 4.3. Preparation of Aqueous Solutions of Amino Acids at 2 mM

Two hundred micromoles of amino acid were thoroughly mixed with distilled water, in a 100 mL–volumetric flask. This solution is a 2 mM aqueous solution of amino acid.

In terms of amino acids, available in monohydrochloride form (namely l-arginine HCl, l-cysteine HCl, l-histidine HCl, and l-lysine HCl), they were dissolved in a 2 mM NaOH solution to afford the same concentration of 2 mM.

### 4.4. Preparation of Aqueous Solutions of Amino Acids at 0.25–16 mM

3.2 Millimoles of amino acid were thoroughly mixed with distilled water in a 200 mL volumetric flask. This solution is a 16 mM stock solution of amino acid. Again, for amino acids that available in monohydrochloride form, they were dissolved in a 16 mM NaOH solution, to afford the same concentration of 16 mM.

Other concentrations were prepared by dilution of the 16 mM stock solution with distilled water to afford concentrations of 0.25, 0.5, 1, 2, 4, and 8 mM, respectively.

### 4.5. Bioassay for Seed Germination and Seedling Growth

Similar to our previous work [44], a small glass vial (4.5 × 2 cm), lined with germination paper, was filled with a 0.5 mL aqueous solution of amino acids. Ten seeds of Chinese amaranth (or barnyard grass) were then placed in the vial, followed by sealing the vial with Parafilm^®^. All prepared vials were kept at 28–30 °C, in a conditioned chamber (Climacell 707, Munich, Germany), with a photoperiod of 12 h and a relative humidity of 80%. After period of 7 days, germinated seeds were counted, and shoot and root lengths were measured. The percent inhibitions were calculated with this equation:

Inhibition (% of control) = 100 − ((amino acid/control) × 100)
(1)

## 5. Conclusions

In summary, herbicidal activity of 22 common α-amino acids has been investigated. Weed control property depended on several factors, including type of enantiomer, chemical structure (substituent), and applied concentrations of the active compounds, as well as species and organs of the tested plants. Comparative studies of d-isomer, l-isomer, and d:l-mixture revealed a sign of enantioselective and synergistic toxicity. Also, type of substituents significantly affected the activity, in which amide (for glutamine), thioether (for methionine), and heteroaromatic (for tryptophan) suppressed plant development the most. d-tryptophan caused leaf breaching in barnyard grass, indicating the mode of action of the compound, which could be the interference of pigment biosynthesis of the plant. Roots were more sensitive to chemicals than shoots. When compared between the two tested species, Chinese amaranth and barnyard grass, the symptom of injury was different. Overall, the current finding would be crucial in the research and development of natural herbicides. Also, the synergistic property will benefit both herbicide manufacturers and farmers, as racemic mixtures are cheaper and easier to make than pure forms. Due to the low production cost, farmers would be easily access to the amino acid herbicides.

## Data Availability

Not applicable.

## Sample Availability

Samples of the 22 amino acids are available from the authors.
